# Low-Molecular-Weight Seaweed-Derived Polysaccharides Lead to Increased Faecal Bulk but Do Not Alter Human Gut Health Markers

**DOI:** 10.3390/foods10122988

**Published:** 2021-12-03

**Authors:** Ciara D. Bannon, Julia Eckenberger, William John Snelling, Chloe Elizabeth Huseyin, Philip Allsopp, Conall Strain, Priya Ramnani, Roberto Chitarrari, John Grant, Sarah Hotchkiss, Kevin Philp, Ross Campbell, Kieran Michael Tuohy, Marcus J. Claesson, Nigel George Ternan, James S. G. Dooley, Roy D. Sleator, Ian Rowland, Chris I. R. Gill

**Affiliations:** 1 The Nutrition Innovation Centre for Food and Health (NICHE), School of Biomedical Sciences, University of Ulster, Cromore Road, Coleraine, Co., Londonderry BT52 1SA, Northern Ireland, UK; cd.bannon@ulster.ac.uk (C.D.B.); b.snelling@ulster.ac.uk (W.J.S.); pj.allsopp@ulster.ac.uk (P.A.); jsg.dooley@ulster.ac.uk (J.S.G.D.); c.gill@ulster.ac.uk (C.I.R.G.); 2 School of Microbiology and APC Microbiome Ireland, University College Cork, T12 K8AF Cork, Ireland; 116223742@umail.ucc.ie (J.E.); chloe.huseyin@ucc.ie (C.E.H.); m.claesson@ucc.ie (M.J.C.); 3 Moorepark Food Research Centre, Teagasc, Fermoy, Co., P61 C966 Cork, Ireland; catchmentarea1060@gmail.com; 4 Department of Food and Nutritional Sciences, University of Reading, Reading RG6 6AP, UK; praya2k@gmail.com (P.R.); roberto.chitarrari@gmail.com (R.C.); i.rowland@reading.ac.uk (I.R.); 5 Kerry Global Technology and Innovation Centre, Millennium Park, Naas, Co., W91 W923 Kildare, Ireland; Johngrant.cork@gmail.com; 6 CyberColloids Ltd., Carrigaline Industrial Estate, Carrigaline, Co., P43 VR72 Cork, Ireland; sarah@cybercolloids.net (S.H.); kevin@cybercolloids.net (K.P.); ross@cybercolloids.net (R.C.); 7 Nutrition and Nutrigenomics Unit, Research and Innovation Centre, Fondazione Edmund Mach, Via E. Mach 1, 38098 S. Michele all’Adige, TN, Italy; Kieran.tuohy@fmach.it; 8 Department of Biological Sciences, Munster Technological University, Bishopstown, T12 P928 Cork, Ireland; Roy.sleator@mtu.ie

**Keywords:** faecal bulk, seaweed, bacteriome, health benefits

## Abstract

Seaweeds are potentially sustainable crops and are receiving significant interest because of their rich bioactive compound content; including fatty acids, polyphenols, carotenoids, and complex polysaccharides. However, there is little information on the in vivo effects on gut health of the polysaccharides and their low-molecular-weight derivatives. Herein, we describe the first investigation into the prebiotic potential of low-molecular-weight polysaccharides (LMWPs) derived from alginate and agar in order to validate their in vivo efficacy. We conducted a randomized; placebo-controlled trial testing the impact of alginate and agar LWMPs on faecal weight and other markers of gut health and on composition of gut microbiota. We show that these LMWPs led to significantly increased faecal bulk (20–30%). Analysis of gut microbiome composition by sequencing indicated no significant changes attributable to treatment at the phylum and family level, although FISH analysis showed an increase in *Faecalibacterium prausnitzii* in subjects consuming agar LMWP. Sequence analysis of gut bacteria corroborated with the FISH data, indicating that alginate and agar LWMPs do not alter human gut microbiome health markers. Crucially, our findings suggest an urgent need for robust and rigorous human in vivo testing—in particular, using refined seaweed extracts.

## 1. Introduction

Human consumption of seaweeds (macroalgae) is relatively limited in Western societies [1]. By contrast, they are a dietary staple in Asia, with seaweed consumed at up to 5.3 g/day particularly in Japan and Korea [2]. Seaweed contains a diversity of macro- and micronutrients, bioactive compounds, complex dietary fibres, polyphenols, fatty acids, and carotenoids, as well as potentially hazardous levels of iodine and heavy metals, such as arsenic [1,2,3,4,5]. Nonetheless, edible seaweeds have the potential to positively impact human health and indeed there is increasing interest in the health benefits associated with seaweed consumption [1,2,3,4,5], specifically in regard to the alleviation of risk factors associated with non-communicable diseases such as obesity, type 2 diabetes, cardiovascular disease and cancer [1].

It is broadly accepted that Western populations fail to meet the recommended daily dietary fibre intake of 25–38 g/day proposed by the European Food Safety Authority [6] and the US Institute of Medicine [7]. Dietary fibre is associated with a wide range of health benefits including faecal bulking which is important for reducing gut transit time and preventing gut motility issues, such as constipation or diverticulosis [8]. The viscous properties of fibre contribute to delayed gastric emptying and slow the rate of nutrient absorption in the small intestine. This leads to improvements in glycaemic control, in addition to reducing appetite, increasing satiation and lowering food intake [9]. Seaweed has a high fibre content (up to 75% of dry mass) and contains high levels of hydrocolloid fibres with gel-forming and emulsifying properties, such as alginate, agar and carrageenan [1]. Consequently, there is growing interest in the pharmaceutical and nutraceutical sectors in the role of seaweed fibres and bioactives in reducing disease risk.

To date, the majority of seaweed studies have used either in vitro fermentation models or in vivo small animal studies to assess post-treatment changes in microbiota composition and metabolism [10]. The high molecular weight of seaweed-based fibres has been suggested to lower their accessibility and fermentability by gut microbiota [11], and there is evidence to indicate that depolymerisation of seaweed fibre polysaccharides such as alginate may enhance their fermentability [12]. Recently, *Faecalibacterium prausnitzii*, a dominant and prevalent commensal butyrate producer and candidate next-generation probiotic, was shown to be selectively enriched within in vitro faecal batch cultures upon cross-feeding on alginate oligosaccharides (AOS) released by *Bacteroides* species from alginate [13]. Furthermore, reducing the molecular weight through depolymerisation reactions to create novel LMWP derivatives decreases the viscosity and gel-forming properties of the polymers, thereby facilitating their incorporation in a variety of food products. Previously, we showed that low-molecular-weight polysaccharides (LMWPs) derived from agar and alginate bearing seaweeds were readily fermented by gut bacteria in vitro where they yielded increased total short-chain fatty acids (SCFA) levels [14]. These LMWPs thus represent a novel source of prebiotic fibres with the potential to benefit markers associated with human health. The current randomized, placebo-controlled trial tested the impact of alginate and agar LWMPs on faecal weight and other markers of gut health as well as on the composition of gut microbiota in order to validate their in vivo efficacy.

## 2. Materials

### 2.1. Low-Molecular-Weight Polysaccharides (LMWP) Intervention Product

Three separate products were tested in the human intervention study: agar H1CC2013, alginate H1CC2012 and a maltodextrin (placebo) control. The LMWP candidates, agar (H1CC2013) and alginate (H1CC2012), were produced by CyberColloids Ltd. (Carrigaline, Ireland) using free radical degradation with L-ascorbic acid as the depolymerisation method as previously described [14]. The agar and alginate powders were commercially sourced; *Gelidium*-derived agar (Industrias Roko S.A., Llanera, Spain) and Manugel DMB sodium alginate (FMC BioPolymer), respectively. Maltodextrin powder (AVEBE™ MD 14P) was supplied by Avebe Food (Veendam, The Netherlands). The formulation for the drink products was created by CyberColloids Ltd. and drinks were packaged by DrinkPac UK. Each drink (250 mL) contained 8 g (3.2%) of experimental agent (agar H1CC2013, alginate H1CC2012 or maltodextrin). The drinks were water-based (90.5%) and in addition contained 6% sucrose, 0.2% citric acid, 0.05% sodium citrate, 0.02% raspberry flavour, 0.01% sodium benzoate and 0.01% artificial colour. All products were identically presented with opaque packaging and a black opaque drinking straw.

### 2.2. Participants

The study was conducted in 60 participants (30 men and 30 women) aged 35.9 ± 8.6 years, range 19–50 years; body mass index (BMI) 25.8 ± 3.4 kg/m^2^, all non-smokers. All participants were healthy, omnivores, not using prescribed medication, nor regularly consuming dietary supplements (including pro/prebiotic products). The study was conducted with the prior approval of the Ulster University Research Ethics Committee (REC/09/0033) and with the informed consent of participants. The work was in accordance with the Declaration of Helsinki and the trial was registered at www.clinicaltrials.gov (NCT02608983; last accessed on 26 November 2021).

### 2.3. Study Design

**Figure 1:**
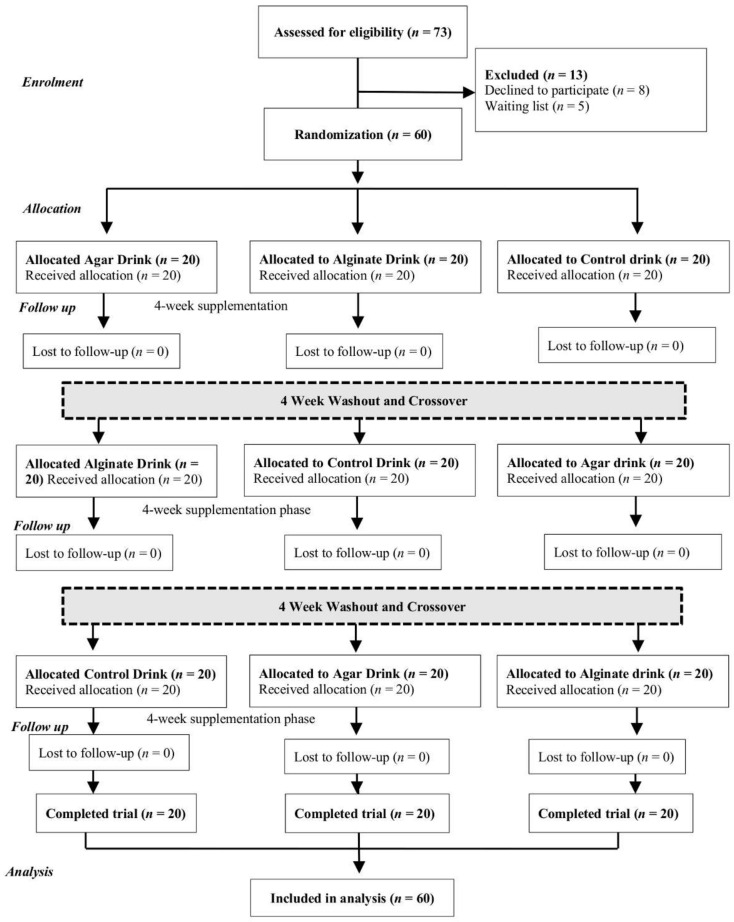
CONSORT flow diagram of participant progress through the study.

The double-blind, randomized crossover design study was reported in accordance with Consolidated Standards for Reporting Trials (CONSORT) guidelines (Figure 1). Participants were randomly assigned to one of three starting treatments: control (Maltodextrin), agar (H1CC2013) or alginate (H1CC2012), during the first phase of the study. Randomization was conducted independently using a computer-generated random sequence [15]. During the treatment phases, participants consumed, daily, a 250 mL drink containing either 8 g of low-molecular-weight agar (H1CC2013) or low-molecular-weight alginate (H1CC2012) for 28 consecutive days (4 weeks), in addition to their normal diet. During the control phase (4 weeks), participants consumed a 250 mL drink (8 g maltodextrin) daily in addition to their normal diet. The three intervention phases were separated by a 4-week washout period during which no dietary intervention products were consumed. For each of the six time-points, pre- and post- of each of three intervention phases, participants provided a faecal sample and a 4-day food diary to monitor habitual diet, which was analysed via NetWISP (v 3.0 Tinuviel, Llanfechell, UK) [16] to generate fibre, fat and carbohydrate (CHO) intakes. Participants also kept a 7-day faecal habit diary during each intervention to monitor the faecal frequency and consistency, abdominal pain (none, mild, moderate or severe), intestinal bloating (none, mild, moderate or severe) and flatulence (none, mild, moderate or severe). Participants were supplied with their allocation (28 days) of test drinks at the beginning of each phase of the trial (week 0 and week 8, week 16) and were asked to return unconsumed drinks to help monitor and aid compliance to the intervention. All participants and researchers were blinded until after completion of the study and data analysis.

### 2.4. Faecal Sample Processing

Faecal samples were collected before and after each treatment phase (weeks 0, 4, 8, 12, 16, and 20). Participants provided fresh faecal samples which were kept at 4 °C and processed within 2 h [17]. Sample consistency was measured using the Bristol faecal form chart [18] and faecal weight and the pH of homogenised faecal samples were measured. Aliquots (1 g and 5 g) of the faecal sample were removed and immediately frozen at −80 °C for subsequent bacterial composition analysis. For faecal water preparation, the faecal sample was mixed 1:1 (weight/volume) with ice-cold sterile phosphate-buffered saline (PBS) and homogenised [17]. The homogenate was ultra-centrifuged at 50,000× *g* at 4 °C for 2 h (Beckman XL 80 Ultracentrifuge), after which the supernatant was filter sterilised (0.22 μm pore diameter, Merck KGaA, Darmstadt, Germany) and stored at −80 °C until required. A separate 5 mL aliquot of faecal homogenate was diluted 1:10 in sterile PBS, further homogenised by vortexing with 3 mm glass beads and then centrifuged in a benchtop microcentrifuge at 1500 rpm for 5 min prior to processing for short-chain fatty acid (SCFA) analysis and Fluorescence in situ hybridization (FISH). Aliquots (1 g and 5 g) of faecal materials described were stored in −80 °C freezers at Ulster University in accordance with the Human Tissue Act 2004 until subsequent analysis.

### 2.5. Short-Chain Fatty Acid Analysis (SCFA)

Short-chain fatty acids (SCFA) acetate, propionate, butyrate, isobutyrate, valerate, isovalerate and caproate were analysed as their silyl-derivatives by gas chromatography [14]. In brief, 1.5 mL of faecal homogenate was centrifuged at 13,000× *g* for 5 min, the supernatant was recovered and an internal butyric acid standard was added (50 µL of a 100 mM stock of 2-ethyl butyric acid). Acids were extracted by addition of 0.5 mL 1 M HCl and 2 mL diethyl ether, followed by vortex mixing for 1 min. The extraction reaction was centrifuged at 3000× *g* for 10 min and the ether layer was recovered and placed in a fresh microcentrifuge tube. A further 1 mL diethyl ether aliquot was added to the aqueous layer and a second extraction was performed. An 800 µL aliquot of the combined ether extracts was then transferred to a screw-capped vial and derivatised using 100 µL of *N*-tert-butyldimethylsilyl-*N*-methyltrifluoroacetamide (MTBSTFA) at 80 °C for 20 min. The reaction mixture was left at room temperature for 48 h to ensure complete derivatisation, following which samples were analysed on a 5890 series II GC system (HP, Crawley, West Sussex, UK) fitted with an SGE-HT5 column (0.32 mm × 25 m × 0.1 µm) (J and W Scientific, *via* Agilent Technologies, Folsom, CA, USA) and a flame ionization detector. The helium carrier gas pressure was set at 10 psi with a split ratio of 10:1. Injector, oven and detector were set at 275 °C, 250 °C and 275 °C, respectively. A 1 µL aliquot of each sample was injected with a run time of 27 min. Peaks were integrated using the Atlas Lab managing software (Thermo Lab Systems, Mainz, Germany). Fatty acid concentrations (mM) were calculated with reference to standard peak areas [14].

### 2.6. Fluorescence In Situ Hybridization (FISH)

The bacterial population diversity in participant samples was screened at each time-point using fluorescence in situ hybridization (FISH) [14]. Samples of faecal homogenate (375 µL) were fixed overnight in 1.125 mL of 4% (*w*/*v*) filtered paraformaldehyde (pH 7.2). The fixed cells were centrifuged at 13,000× *g* for 5 min, washed twice with filtered PBS, resuspended in 300 µL of a (1:1 *v*/*v*) mixture of PBS/ethanol and were stored at −20 °C until required. The hybridization was carried out as previously described [19] using Cy3-labelled oligonucleotide probes (Sigma Aldrich Ltd., Dorset, UK) targeting specific regions of the 16S rRNA gene. The probes used were Eub mix (Eub, EubII, EubIII) [20], Bif164 [21], Lab158 [22], His150 [23], Bac303 [24], Prop853 [25], Fpra645 [26], and Erec482 [23] specific for total bacteria, *Bifidobacterium* spp., *Lactobacillus*/*Enterococcus* spp., *Clostridium perfringens*/*histolyticum* subgroup, *Bacteroides*/*Prevotella* group, Clostridial cluster IX, *Faecalibacterium prausnitzii*, and Clostridial clusters XIVa+b, respectively.

### 2.7. Transepithelial Resistance Assay (TER)

In brief [17], Caco-2 cells (ECACC; Salisbury, UK) were maintained in Minimal Essential Medium (MEM), 10% foetal bovine serum, 2 mM glutamine and 1% penicillin/streptomycin and sub-cultured every 2 days. Cells were seeded onto Transwell polyethylene terephthalate (PET) inserts (BD Biosciences, Bedford, UK), coated with 0.1% rat tail collagen, at a density of 2.5 × 10^5^ cells per insert and grown for 14 days prior to the start of the experiment. At baseline, TER values were in the range of 600–700 Ω·cm^−2^. For the TER assay, faecal water was diluted to 10% (*v*/*v*) in culture medium and added apically, with TER measurements taken at 0, 24 and 48 h. The effect of FW samples was assessed in duplicate and mean values were calculated as% difference from the baseline (0 h) TER value.

### 2.8. Microbiome Analysis

A bacteriome (16S rRNA) population analysis was performed on samples provided by a subset of participants who had provided enduring consent. Samples from five participants (6 time points each; 30 samples in total) consuming the agar treatment were selected based upon their having elevated levels of *Faecalibacterium prausnitzii* as determined by FISH.

#### 2.8.1. DNA Extractions

Genomic DNA was extracted from a ~0.2 g sample of faeces using the QIAamp Fast DNA Faecal Mini Kit (51604) with additional bead beating. The faecal samples were added to a sterile 2 mL microcentrifuge tube containing one 3.5 mm glass bead, 0.1 mL of 1.0 mm zirconia/silica beads and 0.1 mL of 0.1 mm glass beads (Biospec, Bartlesville, OK, USA). 1 mL of InhibitEX buffer was added to each sample prior to disruption by bead-beating in a Mini-Beadbeater-24 (Biospec) at maximum speed (3800 strokes/min). Samples were disrupted for 1 min, followed by incubation on ice for 1 min; the disruption-ice incubation step was then repeated twice before incubation at 95 °C for 5 min. Samples were vortexed for 15 s and then centrifuged at 13,300 rpm for 1 min to pellet the faecal particles. The DNA extraction was then continued as per the kit manufacturer’s instructions. DNA quality was assessed using a Nano-Drop 2000 spectrophotometer (Thermo Scientific, Waltham, MA, USA) and visualised on a 1% agarose gel. DNA concentration was measured using a Qubit™ 3 Fluorometer (Invitrogen™) employing the Qubit™ dsDNA HS Assay Kit. The extracted DNA was stored at −80 °C prior to library preparation.

#### 2.8.2. Amplicon Sequencing

Library preparation for sequencing of the V3-V4 hypervariable region of the 16S rRNA gene was performed following the Illumina (San Diego, CA, USA) recommendations using 15 ng of extracted genomic DNA in a total polymerase chain reaction (PCR) volume of 30 µL. The primers were selected [27] and adapted to contain overhanging nucleotide sequences (Illumina adapters) in combination with the gene-specific sequences and were used at a concentration of 0.2 µM. PCR amplification was performed on a 2720 Thermal Cycler (Applied Biosystems) under the following conditions: 98 °C for 30 s, followed by 25 cycles of 98 °C for 10 s, 55 °C for 15 s, 72 °C for 20 s and a final cycle of 72 °C for 5 min before cooling to 4 °C. Pre- and post-PCR steps were performed in separated and designated areas of the building. The successful generation of PCR amplicons was verified by visualising the PCR product band on a 1% agarose gel.

Post-PCR, amplified products were purified using Agencourt AMPure XP magnetic beads (Beckman-Coulter, Brea, CA, USA) and eluted in 52.5 µL of EB Buffer (Qiagen). After this initial purification, 5 µL of the purified amplicon-PCR product was amplified in a second PCR to add the Illumina barcode sequences to the 16S gene-specific sequences employing the Nextera XT v2 Index primer kit (Illumina). The index PCR conditions were 98 °C for 30 s, followed by 8 cycles of 98 °C for 10 s, 55 °C for 15 s, 72 °C for 20 s and a final cycle of 72 °C for 5 min before cooling to 4 °C. A second purification step with Agencourt AMPure XP magnetic beads was carried out after the index PCR. The final amplicons containing the Nextera indices were eluted in 27.5 µL of EB Buffer, and the concentration of each sample was measured using a Qubit™ 3 fluorometer. Pooled libraries were created by combining 40 ng DNA of each sample. A sample of the final library pool was sequenced at the Teagasc NGS sequencing facility (Teagasc Moorepark, Fermoy, Co. Cork, Ireland) on an Illumina MiSeq generating 2 × 300 bp paired-end reads.

#### 2.8.3. Bioinformatic Analysis

The quality of the resulting 16S rRNA amplicon reads was visualized with FastQC (v 0.11.3) [28] and the reads were consecutively trimmed and filtered using Trimmomatic (v 0.36) [29] ensuring an average phred score of 25 and a minimum length of 50 bases. The remaining reads were imported into the R environment (v. 3.5.1) [30] for further processing with the DADA2 pipeline (version 1.10.1) [31]. After further quality filtering, error correction and chimera removal, the surviving reads were collapsed into amplicon sequence variants (ASVs) and successively exported back into the Linux environment. A second chimera filtering step with the “uchime_ref” command from USEARCH (v. 8.1.1861) in conjunction with the ChimeraSlayer GOLD database (v. 20110519) [32] was performed using both the de novo and reference-based chimera filtering. Finally, the processed sequences were classified (phylum and genus level) utilizing the “classify.seqs” command from the mothur software (v 1.39.5) [33] against the SILVA ribosomal RNA reference database (Release 132) [34]. Only classifications above a bootstrap cut-off of 80% were retained while leaving all other sequences unclassified at that particular rank. Furthermore, only ASVs with a domain classification of Bacteria or Archaea were kept for further analysis.

All downstream analysis was performed in R version 3.5.2. Alpha diversity was estimated as Chao1 richness and Shannon diversity using the R package “iNEXT” (v. 2.0.20) [35]. Wilcox signed-rank tests were used to evaluate the significance before and after each treatment. To investigate the effect of the supplements on overall bacterial composition, beta diversity was evaluated by performing principal component analysis (PCA) on Aitchison distances, which were calculated with the “ALDEX2” (v. 1.14.1) package [36]. ALDEX2 was also used to calculate the differential abundance of taxa before and after dietary intervention. Differences between groups were assessed using permutational multivariate analysis of variance (PERMANOVA) which was implemented using the adonis function from the “vegan” package (v.2.5.6) [37]. All *p* values were adjusted for multiple testing where appropriate, using the Benjamini and Hochberg method [38]. All plots were constructed in “ggplot2” (v. 3.1.0) [39].

### 2.9. Statistical Analysis

The mean values were shown for all participants (*N* = 60) during their supplementation (agar or alginate) phase and during their placebo-control (maltodextrin) phase. Descriptive statistics were used to present participant and faecal characteristics at week 0 by order of treatment allocation (Control-Agar-Alginate; Agar-Alginate-Control; Alginate-Control-Agar). Potential carryover effects were examined to compare measures at the beginning of each intervention phase (week 0, week 8, week 16). Following this, time-by-treatment interaction was examined that compared overall change in biomarker concentrations according to sequence allocation. The differences in the mean were compared using independent or paired-samples *t*-test for normally distributed data, whereas Mann–Whitney *U* or Wilcoxon’s signed-rank tests were used for data that were not normally distributed. All analysis was conducted in duplicate, unless otherwise stated, and the mean values were taken as the final result. For all markers, the results are presented as treatment effects (treatment values minus baseline values) between agar or alginate and the placebo control phase (maltodextrin). To examine treatment effects for the faecal measurements, individual differences between the values before and after each phase for both control and agar or alginate supplementation phases for each participant were calculated. The statistical tests were then carried out on the difference values (after minus before) between treatment (agar or alginate) and control phases (maltodextrin) [40]. For analysis of microbial composition and SCFA levels, paired *t*-tests were used to compare changes in the bacterial populations assessed by FISH from baseline to the end of each treatment for each of the bacterial groups monitored and for SCFAs Significance level was set at *p* < 0.05. All statistical analyses were performed using SPSS, version 25.0 (SPSS Inc., Chicago, IL, USA).

## 3. Results

### 3.1. Participant Characteristics

The participant flow throughout the study is summarized in the CONSORT diagram (Figure 1). Characteristics of the total group of participants at week 0, and in each order of treatment allocation group, are presented in Table 1. Most participants were overweight at the beginning of the trial based on the mean BMI of the group 25.81 ± 3.46 kg/m^2^. No measured characteristics were different between the order of treatment groups. There was no evidence of a carryover effect for any other outcome variable. The habitual dietary intake was not significantly altered by consumption of either control (Maltodextrin), agar (H1CC2013) or alginate (H1CC2012) drink for the four-week intervention period (as outlined in Table 2). The LMWP contribution was excluded from the habitual dietary assessment, as fibre intake was on average ~11 g for all groups, the addition of LMWP (8 g p.d.) represents a ~70% increase in dietary fibre to ~19 g p.d. No change in participant weight or BMI was observed.

### 3.2. Faecal Characteristics

Significant increases in faecal weight resulted from both agar (*p* = 0.002) and alginate (*p* = 0.02) drink consumption, in comparison to the control drink (maltodextrin) treatment (Table 3). This indicated a pronounced faecal bulking capacity for the alginate and agar-based treatments of approximately 30% and 20%, respectively. No significant changes occurred with the faecal frequency following alginate and agar treatments, and minimal changes were observed with the faecal type (Bristol stool chart). Treatment with the agar drink resulted in a small, but significant decrease in faecal pH.

### 3.3. Barrier Function Changes and Faecal SCFA Analysis

Application of 10% faecal water derived from participants who consumed either alginate, agar or control drinks resulted in no significant changes in faecal water activity with respect to Caco2 barrier function (TER) at 24 and 48 h post addition to cells (Table 4).

SCFA analysis revealed that feeding with maltodextrin, agar and alginate all resulted in increased concentrations of SCFAs in faeces (Table 5). Significant increases in levels of propionic acid and butyric acid were observed after feeding with agar, alginate and placebo control (maltodextrin) (Table 5). For maltodextrin, agar and alginate, the propionic acid levels increased from 0.84 to 1.3 mmoles/L (*p* = 0.006), 0.86 to 1.18 mmoles/L (*p* = 0.001), and 0.96 to 1.29 mmoles/L (*p* = 0.003), respectively.

Butyric acid levels increased from 0.97 to 1.57 mmoles/L (*p* < 0.001), 0.82 to 1.44 mmoles/L (*p* < 0.001), and 0.97 to 1.57 mmoles/L (*p* < 0.001) following consumption of control, agar and alginate drinks respectively. No significant changes were observed for acetic acid, isobutyric acid, valeric acid, isovaleric acid concentrations for any of the intervention drinks, while consumption of the alginate drink resulted in a significant decrease in levels of caproic acid. However, maltodextrin consumption resulted in a significant increase in acetic acid concentrations, from 1.67 to 2.03 mmoles/L (*p* < 0.05).

### 3.4. Assessment of Bacteriome Changes Using FISH and 16S Sequence Analysis

#### 3.4.1. Fluorescence In Situ Hybridization (FISH) Analysis

The major bacterial groups within the faecal microbiota were enumerated using FISH [14]. Bacterial populations post-intervention were compared to baseline pre-intervention populations for all treatment groups (Table 6). For the agar treatment, a significant increase in *Faecalibacterium prausnitzii* abundance from log_10_ 9.81 to 9.91 (*p* = 0.04) was observed. A similar magnitude of increase in *F. prausnitzii* was observed for the alginate treatment (log_10_ 9.90 to 9.98), though this was not statistically significant. *Clostridium histolyticum* numbers exhibited a non-significant (*p* = 0.08) decrease from log_10_ 7.79 to 7.72 in treatment samples when compared with the baseline. No other bacterial taxa showed any significant changes after feeding with agar for 28 days. With alginate treatment, a significant decrease in lactobacilli numbers was observed from log_10_ 7.85 to 7.72 (*p* = 0.05) compared to baseline. For the *Atopobium* group, a non-significant (*p* = 0.08) decrease in bacterial numbers from log_10_ 9.30 to 9.21 was observed. None of the other bacterial groups showed any significant changes after feeding with alginate (Table 6).

For the control treatment (maltodextrin) a decrease in bacterial numbers for the *Clostridium histolyticum* group from log_10_ 7.78 to 7.71 (*p* = 0.03) was observed. Bifidobacterial numbers showed a non-significant (*p* = 0.1) increase from log_10_ 9.16 to 9.25. None of the other bacterial groups showed any significant changes after feeding with control maltodextrin.

#### 3.4.2. Bacteriome Sequencing

**Figure 2:**
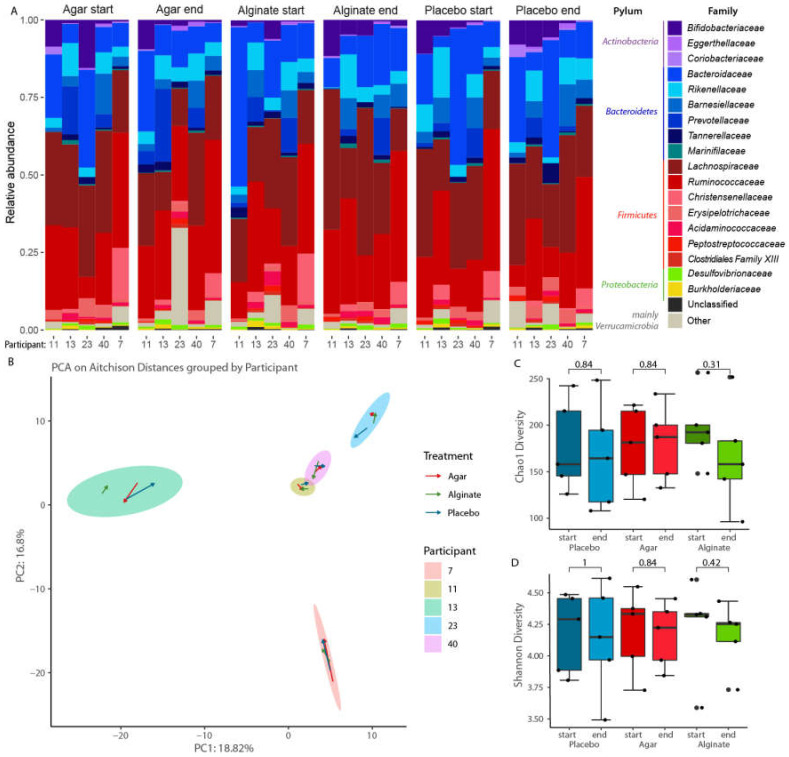
(**A**) Taxonomic composition at family level of each sample ordered vertically by phylum and horizontally by dietary intervention and participant. (**B**) Principal component analysis based on Aitchison distances on all ASVs present in at least two samples grouped by patient. The arrows indicate the shift of the microbial composition after each treatment. Comparison of (**C**) Chao1 diversity and (**D**) Shannon diversity before and after each dietary intervention.

To assess the effects of treatments toward bacteria, 16S rRNA sequencing analysis was performed on 30 selected faecal samples that showed increased *F. prausnitzii* levels following FISH analysis. The 16S rRNA V3-V4 region of 30 samples from 5 participants (3 male, 2 female) was amplified, sequenced and subjected to quality and chimera filtering, resulting in a mean of 14,039 (95% CI: 13,318–14,760) usable reads per sample. The processed reads were collapsed into 1263 ASVs, of which 668 were present in at least 2 samples, and analysed further. Further microbiome analysis from this cohort subsection confirmed that no significant changes were observed after alginate or agar treatments (Figure 2A,B). Differential analysis of taxa before and after the consumption of either agar, alginate or maltodextrin showed no significant change at phylum or family level (Figure 2A, Supplementary Tables S1 and S2).

When considering the overall faecal microbiota composition, the inter-personal differences between the microbial communities were higher than any effect caused by any of the three dietary interventions. Permutational multivariate analysis of variance showed no significant difference in bacterial composition observed before or after the intake of either supplement (Figure 2B). The shifts in the faecal gut microbiota varied not only in strength but also in direction for each intervention and participant. Furthermore, no significant differences in Chao1 diversity or Shannon diversity were observed between the groups (Figure 2C,D).

## 4. Discussion

The majority of research investigating the health benefits associated with fibre and/or its fermentation properties has predominantly focused on cereal and vegetable-derived plant fibre [41]. Here, we report the first in vivo assessment of the effects of depolymerised alginate and agar on gut health. A major finding from the present study was the increase in stool weight in subjects consuming LWMP from agar and alginate, presumably as a consequence of increased water capture [42]. This observation aligns with the work of De Vries et al. [41], who conducted a systematic review of the literature on the effects of cereal, fruit and vegetable fibres on faecal weight and transit time. Their linear regression analyses showed that increased fibre intake had little effect on regularity in individuals with a transit time of <48 h but that, at 8 g p.d., stool bulk increased by between 15% and 35% across all types of fibre. While greater amounts of plant fibre contributed to greater bulking, the increases observed at 8 g p.d. are broadly in line with what we observed in the current work with seaweed-derived LMWP. Given that stool bulk is important for the regulation of normal gut function [43,44] and is also correlated with lowering the risk of colon cancer [45], our investigation demonstrates that increased stool bulk is a key potential health benefit of the addition of depolymerised agar and alginate to foods. Increased dietary fibre has also been found to reduce several other non-communicable diseases associated with the Western lifestyle/developed world including obesity, cardiovascular disease and type 2 diabetes [46] and in a recent review, Corino et al. [47] evaluated dietary seaweed polysaccharide prebiotic supplementation in pigs with respect to their potential for positive gut health and microbiota composition modulation. Overall, supplementation with brown seaweed polysaccharides represented a valid strategy to positively modulate microbiota.

We have previously published evidence that in vitro faecal fermentation of whole brown seaweeds and their extracted complex polysaccharide components increased the production of acetate, propionate, butyrate, and total SCFAs [3]. In the present human intervention study, there were no significant increases in faecal SCFAs detected after agar or alginate LMWP consumption. This is consistent with many dietary fibre supplementation studies since up to 95% of microbiota-derived SCFAs are rapidly absorbed in the colon, resulting in decreasing concentrations from the proximal to the distal colon. Therefore, only a minor fraction of SCFAs (about 5%) is actually excreted in faeces [48]. Consequently, it seems likely that the lack of effect of agar and alginate LMWP on faecal water-induced barrier function (TER) was due to low faecal SCFA concentrations since the latter are important determinants of faecal water TER activity [49]. For example, in a previous in vitro study, we showed that faecal water containing substantial levels of SCFA increased barrier function (TER) in a gut epithelium model [17].

Seaweeds are a rich source of components that may exert advantageous effects on the mammalian gut microbiota through the improvement of bacterial abundance and diversity. A recent comprehensive review of in vitro and in vivo studies by Shannon and colleagues [50] considered the potential therapeutic potential of seaweed-derived polysaccharides, peptides and polyphenols to modulate the gut microbiota through diet. They reported that, in several studies, these components could act as prebiotics and positively modulate the gut microbiota [50]. In the current work, we observed modest changes in the levels of quantified bacteria in stool samples (FISH analysis) following agar and alginate treatments, including a significant increase in *Faecalibacterium prausnitzii* in subjects consuming agar LMWP. *Faecalibacterium prausnitzii* is a highly abundant human gut microbe in healthy individuals and constitutes a biomarker of a healthy gut, being associated with anti-inflammatory properties [51]. Subsequent sequencing analysis of a subset of stool samples (30 samples, 5 participants) with elevated levels of *F. prausnitzii*, showed no significant effects on gut microbiota at the phylum and family levels, an observation which could be due to the relatively low number of samples sequenced.

Recently, Mizuno and co-workers [52] demonstrated that enteral feeding for 4 weeks with an alginate formula (~15 g fibre/day) in the elderly (*N* = 11) altered gut microbiota composition—albeit to a limited extent—with a significant increase (*p* = 0.039) in *Clostridium* cluster XI bacteria, but not in *Bacteroides*, *Bifidobacterium* or *Lactobacillales* compared to baseline. Faecal concentrations of SCFAs were, likewise, not affected but circulating concentrations were significantly increased. Furthermore, faecal pH was lowered and faecal consistency (*p* = 0.044), but not stool frequency, improved. These modest effects on gut microbiota and faecal characteristics are consistent with the limited effects observed for LMWP alginate and agar products in this study, suggesting a limited prebiotic potential. Sakai and co-workers [53] demonstrated that an oral preparation of fucoidan (~1.6 g/d, 12 weeks)—a high molecular weight seaweed polysaccharide—led to alterations of gastrointestinal function in a diabetic population (*N* = 30), including increased faecal frequency. However, faecal weight was not measured, in contrast to the present work with a low-molecular-weight polysaccharide extract, in which we observed increased faecal weight but no significant changes in stool frequencies.

## 5. Conclusions

In this randomised, placebo-controlled trial, we observed significant increases of approximately 30% and 20% in faecal weight after consumption of drinks supplemented with alginate and agar polysaccharide preparations, respectively, indicating a potential as functional food ingredients. However, there was little evidence for benefits with respect to other gut health endpoints, such as stool frequency, microbiota composition, SCFA concentration and epithelial barrier function. This may be due in part to the lack of complexity that would be found in a crude seaweed preparation. Thus, as indicated by Strain and colleagues [5], additional in vivo human studies are required to evaluate the potential of crude seaweed-derived extracts that contain a rich mix of polysaccharide substrates to modulate the microbiota and exert a prebiotic effect.

## 6. Research Highlights

- The first in vivo study to investigate the prebiotic potential of alginate and agar in the human gut.
- Low-molecular-weight seaweed-derived polysaccharides lead to increased faecal bulk.
- No demonstrable alteration of human gut health markers—microbiota, barrier function (transepithelial resistance) or faecal short-chain fatty acid (SCFA) levels.
- The need for additional focused human in vivo investigative studies using crude polysaccharide-rich seaweed extract mixtures.

## Figures and Tables

**Table 1:** Participant characteristics at study commencement (week 0) by allocation to order of treatment.

Measures	Total Group (*N* = 60)	Order of Treatment		
		Control-Agar-Alginate (*n* = 20)	Agar-Alginate-Control (*n* = 20)	Alginate-Control-Agar (*n* = 20)
				
Height (m)	1.71 ± 0.1	1.69 ± 0.08 ^a^	1.69 ± 0.08 ^a^	1.74 ± 0.09 ^a^
Weight (kg)	72.57 ± 10.76	73.72 ± 13.46 ^a^	73.01 ± 11.12 ^a^	81.04 ± 14.82 ^a^
BMI (kg/m^2^)	25.81 ± 3.46	25.67 ± 4.01 ^a^	25.28 ± 2.76 ^a^	26.47 ± 3.29 ^a^
Faecal weight (g)	82.3 ± 71.1	91.23 ± 84.49 ^a^	82.7 ± 68.55 ^a^	84.0 ± 70.49 ^a^
Faecal pH	6.86 ± 0.64	6.71 ± 0.65 ^a^	7.08 ± 0.61 ^a^	6.8 ± 0.63 ^a^
Faecal frequency (7 d)	11.83 ± 8.14	11.55 ± 7.12 ^a^	13.3 ± 11.2 ^a^	9.3 ± 4.31 ^a^
Faecal type (median)	4	4.5	3.5	4

Difference in means (± SD) between allocation to order of treatment. Values sharing a common superscript are not significantly different, *p* < 0.05 (*t*-test, Mann–Whitney U test).

**Table 2:** Anthropometric measurements and habitual dietary intake by intervention.

Treatment		Baseline	Post-Treatment	Change (Post-Pre)
**Control Drink**	Height (m)	1.71 ± 0.1	1.71 ± 0.1	0 ^a^
(*N* = 60)	Weight (kg)	75.7 ± 14.1	75.6 ± 14.4	−0.1 ^a^
	BMI (kg/m^2^)	25.7 ± 3.5	25.7 ± 3.5	0 ^a^
	Energy (kcal)	1860.4 ± 578.5	1798.6 ± 500.2	−61.8 ^a^
	Energy (kj)	7783.8 ± 2420.4	7525.2 ± 2092.8	−258.6 ^a^
	Protein (g)	74.3 ± 27.7	73.9 ± 19.3	−0.4 ^a^
	Total Fat (g)	68.3 ± 30.1	65.9 ± 22.5	−2.3 ^a^
	CHO (g)	211.3 ± 64.9	209.5 ± 69.6	−1.8 ^a^
	Fibre (g)	10.9 ± 4.3	11.9 ± 5.4	1.0 ^a^
**Alginate Drink**	Height (m)	1.71 ± 0.1	1.71 ± 0.1	0 ^a^
(*N* = 60)	Weight (kg)	76 ± 14.2	75.9 ± 14.3	−0.1 ^a^
	BMI (kg/m^2^)	25.8 ± 3.6	25.9 ± 3.6	0.1 ^a^
	Energy kcal	1917.0 ± 506.5	1867.7 ± 653.9	−49.4 ^a^
	Energy (kj)	8020.9 ± 2119.1	7814.4 ± 2735.7	−206.5 ^a^
	Protein (g)	76.1 ± 21.7	75.4 ±28.0	−0.8 ^a^
	Total Fat (g)	71.3 ± 24.8	69.7 ± 31.5	−1.6 ^a^
	CHO (g)	218.0 ± 67.2	207.6 ± 64.4	−10.4 ^a^
	Fibre (g)	11.9 ± 4.9	11.4 ± 4.9	−0.5 ^a^
**Agar Drink**	Height (m)	1.71 ± 0.1	1.71 ± 0.1	0 ^a^
(*N* = 60)	Weight (kg)	76.0 ± 14.3	75.8 ± 14.4	−0.2 ^a^
	BMI (kg/m^2^)	25.8 ± 3.5	25.8 ± 3.6	0 ^a^
	Energy kcal	1838 ± 564.8	1933.4 ± 475.0	−253.0 ^a^
	Energy (kj)	7683.6 ± 2363.1	8089.3 ± 1987.3	−405.7 ^a^
	Protein (g)	75.6 ± 30.0	79.3 ± 20.9	3.7 ^a^
	Total Fat (g)	68.0 ± 27	72.4 ± 22.7	4.4 ^a^
	CHO (g)	205.9 ± 68.7	221.0 ± 74.3	15.1 ^a^
	Fibre (g)	11.0 ± 3.6	11.9 ± 5.2	0.9 ^a^

Response to treatment (after–before) sharing a common superscript are not significantly different compared to control. Wilcoxon signed-ranked test *p* < 0.05. LWMP fibre (8 g, p.d.) was excluded from fibre habitual diet assessment.

**Table 3:** Faecal characteristics resulting from consumption of control, agar, and alginate drink(s).

Treatment	Faecal Weight (g) Change		
	Baseline	Treatment	(Treat-Base Values)
**Control Drink**	82.3 ± 71.1	64.2 ± 48	−18.2 ^a^
**Agar Drink**	75.1 ± 58.7	84.8 ± 52.1	9.7 ^b^
**Alginate Drink ^‡^**	67.3 ± 57.8	68.7 ± 52	1.2 ^b^
**Faecal pH**			
**Control Drink ^‡^**	7.0 ± 0.7	7.2 ± 0.5	0.2 ^a^
**Agar Drink**	7.1 ± 0.6	6.9 ± 0.5	−0.2 ^b^
**Alginate Drink**	6.9 ± 0.6	7.1 ± 0.6	0.2 ^a^
**Faecal Frequency (per week)**			
**Control drink ^†^**	9.8 ± 5.1	10.2 ± 6.4	0.0 ^a^
**Agar Drink ^†^**	11.3 ± 7.7	10.9 ± 5.7	−0.7 ^a^
**Alginate Drink ^¥^**	10.5 ± 5	10.2 ± 4.2	0.3 ^a^
**Faecal type (Scale 1–7; median value)**			
**Control Drink**	4	3	
**Agar Drink**	4	4	
**Alginate Drink**	4	3	

Response to treatment (after-before) sharing a common superscript are not significantly different compared to control. Wilcoxon signed-ranked test *p* < 0.05. ^‡^ Change reported in *n* = 59, ^†^ Change reported in *n* = 51, ^¥^ Change reported in *n* = 46.

**Table 4:** Effect of consumption of control, agar, and alginate drink(s) on faecal water bioactivity.

	Control Drink		Agar Drink		Alginate Drink	
Faecal Water Activity *	Baseline	Treatment	Baseline	Treatment	Baseline	Treatment
**Barrier function (24–0 h)**	111 ± 12 ^a^	111 ± 12 ^a^	114 ± 13 ^a^	111 ± 12 ^a^	111 ± 15 ^a^	112 ± 11 ^a^
**Barrier function (48–0 h)**	106 ± 13 ^a^	107 ± 12 ^a^	109 ± 20 ^a^	105 ± 13 ^a^	107 ± 20 ^a^	106 ± 14 ^a^

* Faecal water bioactivity (barrier function) values represent percentage change in activity after addition of faecal water (10% *v*/*v*) for 24 h and 48 h. Values are normalised to average TER (Ω cm^−1^) of Caco2 cells at 0 h. Values sharing a common superscript across rows are not significantly different. Wilcoxon signed-ranked test *p* < 0.05.

**Table 5:** Effects of consumption of control, agar, and alginate drink(s) on faecal short-chain fatty acid concentration.

SCFA	Control Drink		Agar Drink		Alginate Drink	
(mmoles/L)	Baseline	Treatment	Baseline	Treatment	Baseline	Treatment
**Acetic acid**	1.67 ± 0.93	2.03 ± 1.29	1.66 ± 1.11	1.91 ± 1.15	1.87 ± 1.12	2.09 ± 1.41
**Propionic acid**	0.84 ± 0.59	1.30 ± 0.57 ^a^	0.86 ± 0.60	1.18 ± 0.45 ^a^	0.96 ± 0.63	1.29 ± 0.53 ^a^
**Butyric acid**	0.97 ± 0.62	1.57 ± 0.57 ^a^	0.82 ± 0.54	1.44 ± 0.50 ^a^	0.97 ± 0.62	1.57 ± 0.57 ^a^
**Isobutyric acid**	0.02 ± 0.12	0.01 ± 0.01	0.01 ± 0.02	0.05 ± 0.16	0.03 ± 0.10	0.04 ± 0.16
**Valeric acid**	0.03 ± 0.14	0.01 ± 0.04	0.01 ± 0.06	0.03 ± 0.1	0.03 ± 0.9	0.03 ± 0.16
**Isovaleric acid**	0.01 ± 0.03	0.01 ± 0.07	0.01 ± 0.02	0.03 ± 0.12	0.01 ± 0.05	0.01 ± 0.07
**Caproic acid**	0.03 ± 0.10	0.04 ± 0.15	0.06 ± 0.14	0.03 ± 0.13	0.06 ± 0.12	0.01 ± 0.02 ^a^

^a^: Treatment significantly different from baseline values (*t*-test, *p* < 0.05).

**Table 6:** Faecal bacterial numbers as determined by fluorescence in situ hybridization for sixty participants over the trial period.

	Control Drink		Agar Drink		Alginate Drink	
Bacterial Group	Baseline	Treatment	Baseline	Treatment	Baseline	Treatment
***Total Bacteria***	10.73 ± 0.21	10.75 ± 0.26	10.74 ± 0.23	10.76 ± 0.22	10.76 ± 0.27	10.74 ± 0.20
***Bacteroides* spp.**	10.07 ± 0.26	10.07 ± 0.33	10.03 ± 0.26	10.06 ± 0.36	10.02 ± 0.30	10.04 ± 0.32
***Eubacterium rectale***	9.81 ± 0.24	9.88 ± 0.33	9.81 ± 0.28	9.90 ± 0.30 ^b^	9.86 ± 0.32	9.89 ± 0.31
**subgroup**						
***Bifidobacterium* spp.**	9.16 ± 0.38	9.25 ± 0.37 ^b^	9.16 ± 0.35	9.20 ± 0.32	9.17 ± 0.36	9.20 ± 0.35
***Atopobium* spp.**	9.27 ± 0.37	9.28 ± 0.35	9.31 ± 0.31	9.27 ± 0.37	9.30 ± 0.33	9.21 ± 0.35 ^b^
***Clostridium histolyticum histolyticum* subgroup**	7.78 ± 0.19	7.71 ± 0.21 ^a^	7.79 ± 0.21	7.72 ± 0.23 ^b^	7.78 ± 0.21	7.73 ± 0.18
**Lactobacilli/Enterococci**	7.83 ± 0.37	7.74 ± 0.46	7.82 ± 0.39	7.84 ± 0.48	7.85 ± 0.36	7.72 ± 0.40
**Propionibacteria**	9.80 ± 0.31	9.81 ± 0.34	9.82 ± 0.26	9.79 ± 0.28	9.89 ± 0.26	9.83 ± 0.30
***Faecalibacterium prausnitzii***	9.84 ± 0.29	9.88 ± 0.21	9.81 ± 0.24	9.91 ± 0.28 ^a^	9.90 ± 0.22	9.98 ± 0.25

Bacterial counts in faecal samples are presented as mean log_10_ cells/g faeces: ^a^: Treatment significantly different from baseline values (*t*-test, *p* < 0.05); ^b^: Treatment borderline different from baseline values (*t*-test, *p* = 0.05–0.10).

## Data Availability

The data presented in this study are available on request from the corresponding author (NGT). The data are not publicly available due their containing information that could compromise the privacy of research participants.

## Supplementary Materials

The following are available online at https://www.mdpi.com/article/10.3390/foods10122988/s1, Table S1: Differential abundance analysis with ALDEX2 on family level, Table S2: Differential abundance analysis with ALDEX2 on phylum level.

## Abbreviations

FISH	Fluorescence in situ hybridization
LMWPs	Low-molecular-weight polysaccharides
MTBSTFA	*N*-tert-butyldimethylsilyl-*N*-methyltrifluoroacetamide
SCFA	Short-chain fatty acids
TER	Transepithelial resistance assay
